# Extruded Cassava-Based Corn Analogs Improve Nutrient Digestibility, Gut Health, Intestinal Histology, and Egg Performance in Late-Phase Laying Hens

**DOI:** 10.3390/ani16213418

**Published:** 2026-10-29

**Authors:** Wirot Likittrakulwong, Manatsanun Nopparatmaitree, Payungsuk Intawicha, Sukree Yoosuk, Juan J. Loor, Tossaporn Incharoen

**Affiliations:** 1 Faculty of Food and Agricultural Technology, Pibulsongkram Rajabhat University, Phitsanulok 65000, Thailand; wirotliki@psru.ac.th; 2 Faculty of Animals Science and Agricultural Technology, Silpakorn University, Phetchaburi IT Campus, Phetchaburi 76120, Thailand; nopparatmaitree_m@silpakorn.edu; 3 School of Agriculture and Natural Resources, University of Phayao, Phayao 56000, Thailand; payungsuk.in@up.ac.th; 4 Division of Animal Production Technology, Maejo University, Phrae Campus, Phrae 54140, Thailand; sukree@mju.ac.th; 5 Department of Animal Sciences, University of Illinois, Urbana, IL 61801, USA; jloor@illinois.edu; 6 Faculty of Agriculture, Natural Resources and Environment, Naresuan University, Phitsanulok 65000, Thailand

**Keywords:** extrusion processing, cassava-based corn analog, nutrient digestibility, intestinal morphology, laying performance, late-phase laying hens, volatile fatty acids

## Abstract

This study evaluated extruded cassava-based corn analogs as a dietary replacement for yellow corn in laying hens. A total of 180 Hy-Line Brown hens (60 weeks old) were allocated to four dietary treatments for 10 weeks. Treatments included a standard corn diet (T1), 100% non-extruded analog (T2), 50% extruded analog (T3), and 100% extruded analog replacement (T4). Thermomechanical extrusion significantly increased starch gelatinization to 88.50% and decreased water activity to 0.42. Hens fed the T4 diet achieved the highest apparent ileal starch digestibility (92.1%) and digestible energy (3063 kcal/kg). The T4 group also exhibited increased jejunal villus height compared to the T2 group. Metabolically, this coincided with elevated circulating serum triglycerides and total cholesterol, without disrupting glucose homeostasis. Consequently, the T4 group achieved the highest average egg weight, while maintaining egg mass, yolk weight, and feed conversion ratio at levels statistically comparable to the T1 control. In contrast, the T2 diet resulted in lower starch digestibility, reduced jejunal villus height, and increased cecal volatile fatty. Complete replacement with extruded cassava-based analogs maintains nutrient assimilation and productive performance relative to standard corn. This strategy provides a viable alternative to diversify global feed ingredient sourcing.

## Simple Summary

Yellow corn is the primary energy source in poultry diets. This study corn analogs made from cassava and agro-industrial byproducts, using thermo-mechanical extrusion to improve digestibility. These analogs partially or completely replaced corn in late-phase laying hen diets. Hens fed the 100% extruded analog diet maintained productivity comparable to a standard corn diet. The extruded analog improved starch and energy digestibility and was associated with increased jejunal villus height. This resulted in greater average egg weight and yolk weight, while maintaining egg mass and feed conversion ratio equivalent to the control diet, effectively preventing the performance decline seen with non-extruded analogs. Additionally, extrusion lowered feed water activity, which may support feed stability. Ultimately, extruded cassava-based corn analogs provide an effective nutritional strategy to maintain laying performance and diversify feed ingredient sourcing.

## 1. Introduction

Yellow corn remains an important energy source in global poultry diets, supplying up to 65% of total metabolizable energy [1]. However, severe market volatility and supply chain uncertainties currently compel the industry to seek cost-effective alternatives [2]. Beyond economic pressures, intensive corn cultivation is increasingly discussed in the context of broader regional environmental concerns, particularly in Southeast Asia. The commercialization of upland farming has frequently been linked to practices such as open burning for land preparation [3]. Consequently, the open burning of post-harvest corn residues has been documented as a contributing factor to regional air pollution, including seasonal transboundary haze and elevated PM 2.5 levels [4,5,6]. Exposure to these fine particles poses significant public health risks, primarily by contributing to respiratory issues and degrading the overall quality of life in affected communities. Furthermore, corn production exhibits vulnerable to climate variability due to its high water demand and drought sensitivity during early growth phases [7]. To support the long-term viability of poultry production, exploring resilient and potentially more sustainable carbohydrate sources represents a promising strategy.

In this context, cassava (*Manihot esculenta* Crantz) stands out as a highly viable alternative. Widely recognized for its climate resilience, cassava exhibits robust tolerance to prolonged drought and marginal soil conditions [8]. Unlike water-dependent cereals, it maintains stable yields even at elevated temperatures of up to 35 °C and under severe water scarcity [9]. Additionally, its non-seasonal planting and flexible harvesting timeline provide farmers with crucial agronomic adaptability against unpredictable weather patterns. These unique agronomic traits suggest that cassava could serve as a strategic and potentially ecologically sound alternative to corn in poultry nutrition. Despite its abundant carbohydrate yield, the practical application of cassava root in poultry feeds is significantly restricted by its deficient protein levels, unbalanced amino acid profile, lack of natural pigments, physical dustiness, and the presence of toxic cyanogenic glycosides [10,11]. To rectify these specific nutritional gaps, cassava root can be strategically co-formulated with complementary agro-industrial byproducts to create a nutritionally robust composite matrix. Incorporating corn gluten meal delivers a concentrated source of crude protein and balances the indispensable amino acid profile [12]. Furthermore, broken rice supplies highly digestible starch and supplementary protein, closely mimicking the structural and energy profile of yellow corn [13,14]. The addition of rice bran oil further elevates the overall metabolizable energy density and essential fatty acid content [15], while supplementation with specific pigments compensates for the absence of natural xanthophylls to ensure optimal egg yolk coloration. Thermal and biological interventions, notably boiling, sun-drying, steeping, and fermentation, have been widely validated to detoxify cassava by degrading cyanogenic glycosides to safe thresholds [10]. Numerous studies have validated the use of appropriately processed cassava as a viable energy alternative to corn in poultry nutrition [16,17,18]. Much of this research emphasizes the necessity of enhancing carbohydrate availability and starch utilization. As previously demonstrated, the inclusion of cassava root derivatives in broiler diets can support optimal growth and production parameters whenever the complementary nutrients are precisely synchronized [19]. However, conventional processing techniques such as sun-drying fail to address the physical dustiness and low bulk density of loose composite mixtures. Furthermore, simple drying cannot induce structural macromolecular reorganization or guarantee uniform detoxification, thereby limiting the seamless replacement of whole corn kernels.

To overcome these physical and nutritional bottlenecks, thermo-mechanical extrusion has emerged as a transformative processing technology. By subjecting raw feed ingredients to severe mechanical shear, high pressure, and elevated temperatures within a short residence time, extrusion physically binds loose byproducts into a cohesive, high-density matrix that functions as a structural and functional corn analog [20,21]. Beyond amalgamating the ingredients, this intensive process aggressively volatilizes residual hydrogen cyanide (HCN) and dismantles heat-labile antinutritional factors far more completely than traditional sun-drying. Simultaneously, these combined physical stressors dismantle the native crystalline architecture of starch, driving macromolecular cleavage and extensive gelatinization [22,23]. By breaking down structural and fibrous barriers, extrusion directly exposes the inner carbohydrate core to digestive enzymes while increasing water absorption capacity and enzymatic susceptibility, thereby accelerating overall starch hydrolysis [24]. In poultry nutrition, such targeted structural modifications maximize apparent ileal starch digestibility, optimize metabolizable energy utilization, and enhance feed conversion efficiency [20,25]. The enhanced nutrient digestibility afforded by extrusion becomes particularly crucial as laying hens advance into their late production phase. Beyond peak production, hens experience a natural deterioration in laying performance and egg quality due to the cumulative oxidative stress of sustained oviposition [26]. This age-related regression is heavily driven by a combination of declining reproductive hormones and compromised gastrointestinal function. Furthermore, a marked reduction in the secretion of critical endogenous digestive enzymes, including pancreatic amylase, lipase, and mucosal disaccharidases, severely limits their natural digestive efficiency [27]. In this physiological context, extruded gelatinized starch effectively acts as a pre-digested energy substrate, directly alleviating the metabolic load on the hen’s waning enzymatic capacity.

Applying extrusion technology to formulate extruded cassava-based corn analogs represents a practical strategy to mitigate the physiological limitations inherent to late-phase laying hens. The independent effects of thermal processing and mixed feed formulations are well documented in poultry nutrition. However, the specific impact of using thermo-mechanical extrusion to engineer a complete corn substitute remains poorly characterized. Consequently, this study was conducted with two main objectives. The first aim was to determine the physicochemical properties, color profiles, and water activity of the extruded cassava-based corn analogs. The second aim was to evaluate the biological efficacy of replacing 50% or 100% of dietary yellow corn with these analogs in late-phase diets. To achieve this, we assessed productive performance, egg quality, apparent digestibility, blood glucose, serum biochemistry, cecal volatile fatty acids (VFAs), and intestinal mucosal architecture. We hypothesized that the structural modifications induced by extrusion would significantly enhance starch gelatinization and nutrient utilization. This enhancement would subsequently preserve gut functionality and maintain laying performance, effectively offsetting the age-related decline in digestive capacity.

## 2. Materials and Methods

### 2.1. Processing of Corn Analogs and Dietary Treatment Preparation

Fresh cassava tubers were purchased from local farms in Phitsanulok, Thailand. The roots were thoroughly washed with tap water to remove residual soil. Cleaned tubers were then sliced into chips measuring 1 to 2 cm in thickness. These chips were sun-dried for four to five days until the final moisture content dropped below 12%. Other fundamental ingredients included broken rice, corn gluten meal, rice bran oil, and canthaxanthin. These materials were sourced from commercial suppliers. All raw materials were mechanically pulverized using a hammer mill fitted with a 2.0 mm screen. The entire feed preparation and processing procedure was independently repeated ten times. For each of the ten independent cycles, the ground components were weighed and combined in an electric mixer to achieve a homogeneous basal mash, following the formulation outlined in Table 1. This independently prepared mash was then divided into two distinct processing streams. The first stream was sealed in airtight containers and stored at room temperature, serving as the non-extruded corn analog. Meanwhile, the second stream was subjected to extrusion processing, contributing to the total of ten independent batches of the extruded corn analog. This extrusion step was performed using a laboratory-scale twin-screw extruder (CTE-D22L32, Charoen Tut Co., Ltd., Samut Prakan, Thailand), with the feed mash pre-conditioned to a moisture level of 25 ± 5% using distilled water prior to thermal processing. The extruder barrel temperatures were strictly controlled at 45 °C for the feeding zone, 80 °C for the kneading zone, and 100 °C for the cooking zone. These temperatures were selected to ensure complete starch gelatinization and HCN volatilization while preventing Maillard browning. The internal twin-screw speed was maintained at a constant 300 rpm to provide optimal mechanical shear and material residence time. The extrudate was discharged through a 6.0 mm circular die and immediately sectioned by a rotating die-face cutter operating at 7.0 rpm. The freshly processed products were dried in a forced-air oven at 60 °C for 60 min. This step was necessary to reduce the final moisture content to below 10%. Once cooled to room temperature, the extruded products were packaged in sealed polyethylene bags until required for final feed manufacturing. Lastly, all experimental diets were formulated to be isonitrogenous and isoenergetic. Metabolizable energy equivalence across treatments was established through calculation based on standard nutritional values of the ingredients. The dietary nutrient profiles were balanced to meet or exceed the nutrient requirements specified by the Hy-Line Brown Management Guide [28]. Complete details regarding the feed ingredients and analyzed chemical compositions of the experimental diets are provided in Table 2.

### 2.2. Evaluation of Physicochemical Properties, Color Profiles, and Water Activity

To assess the degree of starch gelatinization, each sample (yellow corn and both unextruded and extruded corn analogs) was analyzed (*n* = 10) using the amyloglucosidase assay outlined by Sá et al. [30]. Furthermore, the water absorption index (WAI) and water solubility index (WSI) were analyzed (*n* = 10) following the methodology of Anderson et al. [31]. Briefly, 2.5 g aliquots were dispersed in 30 mL of distilled water, agitated at 30 °C for 30 min, and centrifuged at 1739× *g* for 10 min. The resulting gel was weighed to determine the WAI, expressed as grams of absorbed water per gram of dry matter. To determine the WSI, the collected supernatant was decanted into pre-weighed aluminum dishes and oven-dried at 105 °C for 4 h. The residual dried solids were weighed, and WSI was calculated and expressed as the mass percentage of dissolved solids relative to the initial 2.5 g dry sample. Sample color was evaluated in ten replicates per treatment according to Bender et al. [32], using an UltraScan VIS spectrophotometer (HunterLab, Reston, VA, USA). Results are expressed in the CIELAB color system (L*, a*, b*). In addition, the water activity of the yellow corn and both unextruded and extruded corn analogs was quantified with a Lab Swift water activity meter (Novasina AG, Lachen, Switzerland) using ten replicates per treatment.

### 2.3. Ethical Approval

All animal management protocols were performed in accordance with institutional ethical guidelines following formal approval from the Animal Care and Use Committee of Naresuan University (Approval No. 69 01 035). The date of approval was 25 May 2026.

### 2.4. Experimental Design and Animal Management

The study followed the ethical guidelines for scientific research established by the National Research Council of Thailand. A total of 180 sixty-week-old Hy-Line Brown laying hens were used in a 10-week feeding trial during late-phase production. The initial average body weight of the birds was 1.85 ± 0.05 kg. Before starting the feeding trial, all hens were subjected to a 7-day adaptation period and were fed the standard control diet to ensure environmental adaptation. Laying hens demonstrating uniform body weights and baseline egg production rates (as detailed were randomly assigned to one of four dietary treatments following a completely randomized design. The first group received a standard corn-based control diet (T1). The remaining groups received diets in which the standard yellow corn was substituted with corn analogs. Specifically, T2 featured a 100% replacement with non-extruded corn analogs. T3 and T4 involved 50% and 100% replacements with extruded corn analogs, respectively. Each treatment consisted of 15 replicate cages, with three hens housed per cage. The cage was considered the experimental unit for all performance and egg quality analyses. The trial was conducted in a closed housing system using evaporative cooling and tunnel ventilation. Each wire cage (43 × 50 × 43 cm) provided adequate stocking density in accordance with animal welfare regulations. Each cage was equipped with a trough feeder and a nipple drinker to allow *ad libitum* access to feed and fresh water. A controlled lighting regimen of 16 h of light and 8 h of darkness (16L:8D) was maintained throughout the trial. Temperature and relative humidity were recorded daily, with the facility maintained at an average temperature of 26.5 ± 1.2 °C and a relative humidity of 65.0 ± 5.0%. Bird health and mortality were monitored daily by trained staff. Upon completion of the 10-week experiment, individual final body weights were recorded for all remaining hens.

### 2.5. Laying Performance and Egg Quality

Total eggs produced, including intact, dirty, and cracked eggs, were collected and weighed twice daily at 07:00 A.M. and 04:00 P.M. to determine average egg weight. Daily hen-day egg production was calculated by dividing the total number of eggs laid per cage by the number of live hens present, multiplied by 100. Daily egg mass was calculated by multiplying the hen-day egg production percentage by the average egg weight. Feed intake was measured and calculated by subtracting feed refusals from the total feed offered. The feed conversion ratio (FCR) was expressed as grams of feed consumed per gram of egg mass. To evaluate egg quality, two intact representative eggs were randomly collected per cage (replicate) on the final two consecutive days of each week. All physical and internal quality parameters measured from these subsampled eggs were averaged within each cage to generate a single mean value per experimental unit for each time point prior to statistical analysis. Egg weight, albumen height, eggshell breaking strength, yolk height, yolk diameter, yolk index, yolk color, and Haugh unit were determined using an automated digital egg tester (DET-6000, Nabel Co., Ltd., Kyoto, Japan). Following automated analysis, the yolk and albumen were separated and weighed individually. To evaluate eggshell parameters, the internal membranes were carefully removed, and the shells were air-dried at room temperature before final weighing. Eggshell thickness was evaluated at three distinct anatomical sites per egg (blunt end, equator, and pointed end) using a digital micrometer (Model 293-240-30, Mitutoyo Corp., Kawasaki, Japan; 0.001 mm accuracy). The average of these three measurements was defined as the mean eggshell thickness.

### 2.6. Euthanasia and Sample Collection

Following the 10-week trial, at 70 weeks of age, one hen per replicate cage representing the mean body weight of that cage was selected, resulting in 15 birds per dietary treatment. These exact same birds were utilized for all concurrent sample collections, including blood, tissues, cecal contents, and ileal digesta. Blood samples (approximately 3 mL) were collected via wing vein puncture and subsequently divided into two distinct aliquots. The first aliquot (~20 µL of whole blood) was immediately analyzed for glucose determination without adding anticoagulants to prevent analytical interference. The second aliquot was allowed to clot in standard blood collection tubes to yield serum for the evaluation of biochemical parameters. Post-blood sampling, hens were humanely euthanized by exsanguination following a two-phase carbon dioxide (CO_2_) stunning protocol (30% CO_2_ for induction, 60% CO_2_ for deep anesthesia). The entire gastrointestinal tract was immediately excised for sampling. To evaluate total VFA profiles, cecal contents from the sampled hens were harvested and pooled. Specifically, individual cecal digesta from three distinct cages within the same treatment were combined into a single composite sample, yielding five independent pooled replicates (*n* = 5) per dietary group. These composite samples were designated as the experimental unit for the VFA analysis. These pooled samples were immediately frozen and stored at −20 °C until further analysis. For histological evaluations, intestinal specimens were collected from five randomly selected hens per treatment (representing one bird from five distinct replicate cages), establishing the individual bird as the experimental unit for these morphological measurements. The small intestine was anatomically divided into three segments: the duodenum (gizzard to pancreatic and bile ducts), jejunum (ducts to Meckel’s diverticulum), and ileum (Meckel’s diverticulum to the ileocecal junction). A transverse tissue section (~3 cm) was excised from the midpoint of each respective segment. The tissue specimens were flushed with 0.1 M phosphate-buffered saline (PBS) to remove luminal contents. For light microscopy, the washed duodenum, jejunum, and ileum sections were fixed in 10% neutral buffered formalin (Sigma-Aldrich, St. Louis, MO, USA) and stored at 4 °C pending histological processing. For scanning electron microscopy, sampling was collected from the jejunum. Jejunal tissues were prepared according to the method described by Incharoen et al. [33]. Briefly, specimens were opened longitudinally along the mesenteric line, washed with 0.1 M PBS (pH 7.4), fixed, post-fixed in 1% osmium tetroxide, and sequentially dehydrated in ethanol prior to analysis.

### 2.7. Blood Glucose and Serum Biochemical Analysis

For blood glucose concentrations, each sample was determined immediately upon collection using a Glucomen Glyco analyzer (A. Menarini Diagnostics, Florence, Italy) with dedicated test strips, following the procedure described by Namted et al. [34]. For serum biochemical profiles, blood samples were centrifuged at 3000× *g* for 15 min at 4 °C to obtain serum, which was aliquoted and stored at −80 °C until analysis. Serum biochemical parameters were determined using an automated clinical chemistry analyzer (Hitachi High-Tech Corp., Tokyo, Japan) and commercial diagnostic kits (Maccura Biotechnology Co., Ltd., Chengdu, China) according to the manufacturer’s protocols. Evaluated parameters included total protein, albumin, creatinine, uric acid, total cholesterol, triglycerides, aspartate aminotransferase, and alanine aminotransferase. Globulin concentration was calculated by subtracting albumin from total protein.

### 2.8. Cecal VFA Quantification

Approximately 1 g of cecal sample was suspended in 9 mL of ice-cold distilled water and homogenized using a Digital Ultra-Turrax T25 (IKA, Staufen, Germany). The homogenate was treated with 0.05 mL of saturated HgCl_2_, 1 mL of 25% H_3_PO_4_, and 0.2 mL of 2% pivalic acid. The mixture was centrifuged at 1000× *g* for 20 min at 4 °C, and a 1.5 mL aliquot of the clear supernatant was collected and stored at −20 °C prior to analysis. Total VFA concentrations were quantified via gas chromatography using an Agilent 7890B system (Agilent Technologies, Santa Clara, CA, USA) according to established conditions [35,36,37]. Final VFA concentrations were determined against calibration curves of authentic standards using a modified calculation method described by Rohde et al. [38].

### 2.9. Intestinal Histological Determination

The preserved intestinal tissues (*n* = 5) were dehydrated in a graded alcohol series, followed by clearing and paraffin embedding. Five-micrometer cross-sections were prepared and subjected to standard hematoxylin and eosin staining. Morphometric observation relied on an OPTIKA B-380 light microscope integrated with OPTIKA Vision Lite software (OPTIKA Microscopes, Bergamo, Italy). For each intestinal segment, 12 well-oriented villi and their associated crypts were measured per bird. These individual measurements were then averaged to obtain a single mean value per bird prior to statistical analysis. Based on the protocol by Incharoen et al. [39], measurements for each segment comprised villus height (VH), crypt depth (CD), the resulting VH:CD ratio, and villus surface area. For the evaluation of epithelial cell changes at the villus apex, designated jejunal samples were evaluated using scanning electron microscopy according to the method described by Incharoen et al. [32]. Prior to lyophilization, tissues were dehydrated through 80%, 90%, and 100% ethanol (120, 60, and 15 min, respectively) followed by three 15 min immersions in tert-butanol. The dried specimens were mounted with conductive carbon paste on aluminum stubs, sputter-coated (Q150R, Quorum Technologies Ltd., Laughton, UK), and visualized at an accelerating voltage of 8 kV (LEO 1455VP, Carl Zeiss, Oberkochen, Germany).

### 2.10. Ileal Digestibility Assay

Apparent ileal digestibility (AID) was assessed using 0.5% titanium dioxide (TiO_2_) as an inert marker in the diets. During the final three days of the 10-week feeding trial, all hen were fed the TiO_2_-supplemented diets to allow for marker equilibration. Feed was provisioned twice daily (07:00 A.M. and 04:00 P.M.) to ensure a controlled intake pattern. Following the humane euthanasia of the selected hens (as described in Section 2.6), the abdominal cavity was promptly incised to isolate the small intestine. Ileal digesta was then harvested by gently expressing the contents of the terminal ileum directly into sterile containers. Ileal digesta from three birds representing three cages within the same treatment were pooled into a single composite sample. This pooling strategy yielded five independent composite samples per dietary group, which were designated as the experimental unit for all digestibility analyses (*n* = 5). These pooled samples were immediately stored at −20 °C to prevent enzymatic activity. Prior to chemical evaluation, both the digesta and experimental feed samples were lyophilized (FreeZone, Labconco, Kansas City, MO, USA). Dried samples were ground through a 0.5 mm screen prior to chemical analysis. Total nitrogen content was determined via the combustion method (Method 990.03) [29] using a TruMac N analyzer (Leco Corp., St. Joseph, MI, USA), with EDTA serving as the calibration standard. Total starch was analyzed using a Megazyme assay kit (Method 996.11) [29]. Gross energy (GE) was measured via an automatic bomb calorimeter (AC-500, LECO Corp., St. Joseph, MI, USA) using a benzoic acid standard. Dietary and digesta TiO_2_ concentrations were determined colorimetrically [40]. AID coefficients for energy, crude protein, and starch were calculated using the indicator method adapted from Aderibigbe et al. [41]. Additionally, ileal digestible energy (IDE; kcal/kg DM) was calculated using the equation adapted from Dao et al. [42]. Both parameters were determined as follows:

AID %=1−TiO2dietTiO2digesta × Nutrient digestaNutrient diet ×100

$$\mathrm{IDE}(\mathrm{kcal}/\mathrm{kg DM})={\mathrm{GE}}_{\mathrm{diet}}-\left[{\mathrm{GE}}_{\mathrm{digesta}}\times \left(\frac{{\mathrm{TiO}}_{2(\mathrm{diet})}}{{\mathrm{TiO}}_{2(\mathrm{digesta})}}\right)\right]$$

where TiO_2(diet)_ and TiO_2(digesta)_ represent the titanium dioxide concentrations in the experimental diets and ileal digesta, respectively; Nutrient_diet_ and Nutrient_digesta_ represent the concentrations of the specific nutrient in the diet and digesta; and GE_diet_ and GE_digesta_ denote the gross energy values of the diets and digesta, respectively. All values are expressed on a dry matter basis.

### 2.11. Statistical Data Analysis

To address the different levels of replication across datasets, the experimental units and sample sizes for each major outcome were assigned as follows: (1) feed physicochemical data used the sample aliquot as the experimental unit (*n* = 10); (2) productive performance and egg quality used the cage as the experimental unit (*n* = 15 cages per treatment), where weekly measurements were averaged over the entire 10-week period to obtain a single mean value per cage; (3) blood biochemistry and individual serum data used the individual sampled bird as the experimental unit (*n* = 15 birds per treatment); (4) cecal volatile fatty acids and apparent ileal digestibility used the pooled composite sample as the experimental unit (*n* = 5 pools per treatment); and (5) intestinal histology used the individual sampled bird as the experimental unit (*n* = 5 birds per treatment). When multiple microscopic observations were recorded for histological assessment, these subsample values were averaged to derive a single representative mean per bird prior to statistical analysis. All data were statistically analysed by one-way analysis of variance (ANOVA) of SPSS Statistics version 31.0 (IBM Corp., Armonk, NY, USA) with a completely randomized design. Upon detection of significant main effects, treatment means were differentiated using Tukey’s honestly significant difference (HSD) test. The alpha level for statistical significance was established at *p* < 0.05. Data are reported as treatment means with the standard error of the means (SEM). All reported SEM values represent the pooled standard error of the treatment mean.

## 3. Results

### 3.1. Physicochemical Properties, Color Profiles, and Water Activity

The extruded corn analogs had a significantly higher degree of gelatinization at 88.50% compared to standard yellow corn at 14.20% and the non-extruded analogs at 16.80% (*p* < 0.001; Table 3). Both the WAI and WSI were significantly elevated in the extruded corn analogs compared with the unextruded treatments (*p* < 0.001). These physicochemical parameters did not differ statistically between the raw yellow corn and the non-extruded corn analog. Colorimetric evaluations indicated consistent visual attributes across all tested feedstuffs. The lightness (L*), redness (a*) and yellowness (b*) did not differ among experimental groups (*p* > 0.05). For the water activity measurement, the extruded corn analog had a significantly reduced water activity level of 0.42 (*p* < 0.001). This measured value was statistically lower than the water activity recorded for both the standard yellow corn (0.58) and the non-extruded corn analog (0.56).

### 3.2. Productive Performance and Egg Quality

The mortality rate was 0% for all dietary treatments throughout the entire experimental period. Additionally, the incidence of dirty and cracked eggs was negligible throughout the experimental period, averaging < 1.0%, and did not significantly differ among the dietary treatments (*p* > 0.05). Dietary replacement of standard yellow corn with extruded or non-extruded corn analogs did not significantly alter initial or final body weights, hen-day egg production, or average daily feed intake across treatment groups (Table 4). However, productivity parameters exhibited clear processing-dependent responses. Incorporation of the unextruded cassava analog (T2) resulted in a noticeable decline in laying performance, yielding the lowest values for average egg weight and egg mass, alongside the highest FCR. Thermo-mechanical extrusion effectively prevented these performance impairments. Specifically, hens fed the 100% extruded analog diet (T4) achieved a significantly greater average egg weight (63.55 g) compared to those fed the T2 (58.92 g) and T1 diets (61.51 g) (*p* < 0.001). For egg mass and FCR, the T4 diet significantly improved performance relative to the unextruded T2 diet (48.99 g/hen/d vs. 44.31 g/hen/d, and 2.14 vs. 2.34, respectively; *p* < 0.05), effectively maintaining these parameters at levels statistically comparable to the T1 control diet (47.01 g/hen/d and 2.24, respectively). Regarding egg quality parameters, yolk weight reached its maximum in the T4 group (17.50 g) and was significantly lower in T2 (15.00 g) (*p* = 0.020). In contrast, whole egg weight, shell weight, shell thickness, shell strength, albumen weight, albumen height, Haugh unit, yolk height, yolk diameter, yolk index, and yolk color did not differ among treatments (*p* > 0.05).

### 3.3. Digestible Energy and Apparent Ileal Nutrient Digestibility

Hens fed the T4 diet achieved the highest IDE at 3063 kcal/kg (Table 5). This value was significantly greater than that of the T2 diet (2950 kcal/kg; *p* = 0.038). Similarly, the T4 group exhibited the highest apparent ileal energy digestibility at 82.9%, which was significantly higher than that of the T2 group (79.9%; *p* = 0.041). Both T1 and T3 groups had intermediate values for both energy parameters. Apparent ileal starch digestibility was highest in the T4 group (92.1%), which was significantly greater compared with the T2 group (85.2%; *p* = 0.015). Apparent ileal protein digestibility did not differ among treatments (*p* = 0.354).

### 3.4. Serum Biochemical Parameters

Blood glucose concentration did not differ among treatment groups (Table 6). However, total cholesterol was highest in hens fed the T4 diet (182.0 mg/dL), reaching levels significantly greater than those in the T2 group (145.0 mg/dL; *p* = 0.029). Serum triglycerides were significantly higher in the T3 group (860.0 mg/dL) compared with the T2 group (750.0 mg/dL; *p* = 0.020). Protein metabolites and renal function markers remained unchanged across treatments (*p* > 0.05). Total protein (5.0 to 5.4 g/dL), albumin (2.0 to 2.2 g/dL), globulin (3.0 to 3.2 g/dL), creatinine (0.3 to 0.6 mg/dL), and uric acid (4.6 to 5.1 mg/dL) did not differ among groups. Similarly, serum activity of hepatic enzymes including aspartate aminotransferase (152.0 to 188.0 U/L) and alanine aminotransferase (17.0 to 22.0 U/L) was not affected by diets (*p* > 0.05).

### 3.5. Cecal VFA Profiles

Dietary substitution with extruded corn analogs significantly altered the cecal VFA concentrations in laying hens (Table 7). In particular, compared with the T2 treatments, the total VFA was significantly lower in the T4 group (93.3 µmol/g (*p* = 0.045). However, there were no significant differences in concentrations of acetic acid, propionic acid, butyric acid, isobutyric acid, valeric acid, and isovaleric acid among the dietary groups.

### 3.6. Villi Morphological Alterations

Dietary replacement with extruded corn analogs significantly influenced jejunal mucosal architecture while maintaining structural integrity in the duodenum and ileum (Table 8). In the jejunum, hens fed the T4 diet achieved the highest VH (985 µm), which was significantly greater than the T2 group (845 µm; *p* = 0.018). In contrast, T2 displayed the deepest jejunal CD (195 µm; *p* = 0.042). As a result, the jejunal VH to CD ratio was significantly lower in T2 (4.35) relative to T1 (5.72) and T4 (6.05) (*p* = 0.013). No significant dietary effects were detected in any morphological parameters of the duodenum or ileum (*p* > 0.05). Across these segments, VH, villus surface area, CD, and the VH:CD ratio remained consistent among the four experimental groups.

### 3.7. Epithelial Apical Surface Using Scanning Electron Microscopy

**Figure 1:**
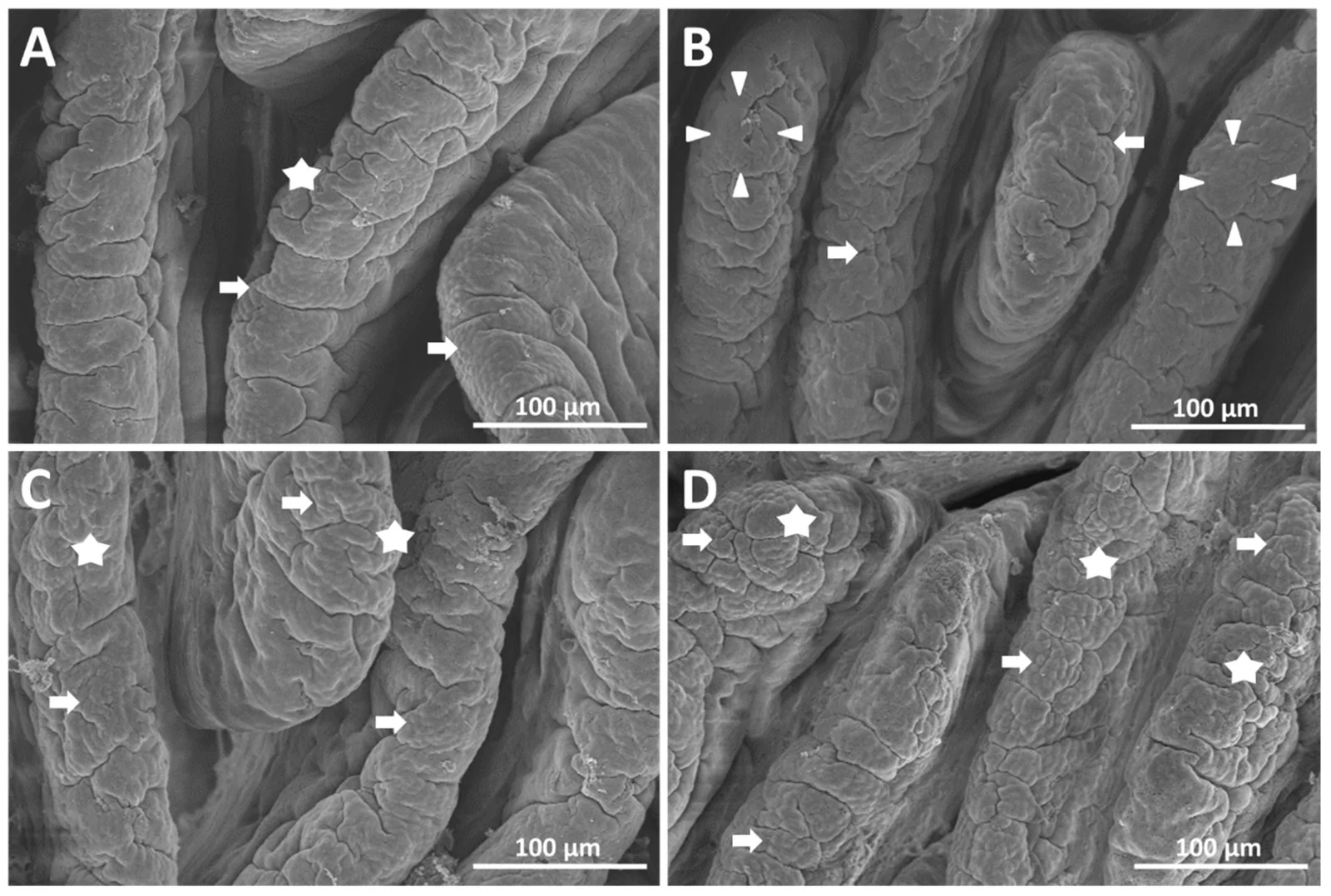
Representative qualitative observations of the apical surface morphology of jejunal villi in laying hens during late-phase production across dietary treatments. Specimens were selected from the jejunal midpoint and visualized across representative random fields. The panels represent hens fed (**A**) a standard corn-based control diet, (**B**) a diet with 100% replacement using non-extruded corn analogs, (**C**) a diet with 50% replacement using extruded corn analogs, and (**D**) a diet with 100% replacement using extruded corn analogs. Morphological features are indicated as follows: arrows highlight protuberant cell areas, stars denote distinct cell clusters, and arrowheads point to flattened cellular regions. Scale bar = 100 µm (300×).

Qualitative scanning electron micrographs of the jejunal villi were evaluated to observe potential microstructural variations in the apical surface across the treatment groups (Figure 1). These images serve as representative observations, with specimens selected from the midpoint of the jejunum and evaluated across random fields of view per treatment group. Hens fed the standard corn control diet (Figure 1A) displayed an organized villous apex with prominent protuberant cell areas and defined cell clusters, producing a densely folded surface contour. In contrast, total replacement with non-extruded corn analogs (T2; Figure 1B) was associated with visually apparent structural alteration. The apical surface in the T2 group was dominated by flattened cellular regions with a visually marked reduction in protuberant cells and active clusters. The inclusion of extruded corn analogs qualitatively preserved microstructural integrity. The T3 group (Figure 1C) exhibited an apparent partial restoration of surface complexity, showing observable protuberant cells and localized cell clusters. Furthermore, the apical surface of villi in the T4 group (Figure 1D) showed dense protuberant cell areas and prominent cell clusters, maintaining a complex, highly folded surface topography.

## 4. Discussion

The pronounced starch gelatinization observed in the extruded cassava-based corn analog confirms the impact of structural modifications on the complex carbohydrate matrix under thermomechanical processing. The synergistic effects of elevated temperatures, high pressure, and intense shear stress completely disrupted the native semi-crystalline architecture of the starch granules [43,44]. This structural reorganization is the direct cause of the significant simultaneous increase in the WAI and WSI of the extrudates [43]. The high-pressure environment within the extruder barrel facilitates the deep penetration of water molecules into the newly exposed amorphous matrix. Within this microenvironment, water acts as a critical plasticizer. It forms new hydrogen bonds with the hydroxyl groups of the starch polymers [45]. Replacing intramolecular bonds with dynamic starch-water interactions significantly enhances the hydration capacity of the matrix. Furthermore, the physical disruption during extrusion also extends to the cleavage of covalent linkages. Mechanical shear induces the partial depolymerization of starch components, fracturing long-chain amylopectin molecules into lower molecular weight dextrins. Combined with the loss of starch granule integrity, these changes modify the rheological properties and overall functionality of the starch [46].

In addition to these structural improvements, maintaining physical quality and long-term storage stability is essential for practical application in the commercial feed industry. Color consistency across all experimental groups demonstrated that the addition of canthaxanthin effectively replicated the visual characteristics of standard yellow corn. Although direct quantification of residual pigment was not performed, the stable color profile suggests that the pigment was not severely degraded during the extrusion process. Heat and oxidation typically degrade canthaxanthin rapidly during conventional processing. However, encapsulating the pigment within a protective matrix, such as soluble soybean polysaccharides, has been reported to aid in color retention and stability [47]. It is plausible that the polysaccharides and starch components of the extruded cassava-based corn analogs formed an encapsulation network within the amorphous carbohydrate matrix during dehydration, which may have contributed to the stability of labile compounds [48]. Additionally, the combination of die-exit flashing and controlled post-extrusion drying substantially reduced the water activity of the samples (0.42). This exceptionally low thermodynamic energy state of water is generally recognized as inhospitable to vegetative microbial growth and fungal proliferation [49]. Consequently, this thermodynamic stability has the potential to safeguard the nutrient-dense feed matrix against mycotoxin synthesis during prolonged warehouse storage, although long-term shelf-life evaluations remain necessary to confirm this [50].

The improvements in apparent ileal starch digestibility, IDE, and overall energy digestibility in hens fed diets containing 100% extruded corn analog directly result from the structural modifications achieved during the extrusion process. The extensive gelatinization of resistant raw starch creates a highly amorphous and pre-digested matrix. This physical transformation increases the available surface area for endogenous enzymes to hydrolyze the substrate. The thermal disruption of the native granular structure into an amorphous state eliminates critical physical barriers. This transformation provides pancreatic α-amylase with unrestricted spatial access to the α-1,4 glycosidic cleavage sites, significantly increasing the kinetic susceptibility of the starch to enzymatic hydrolysis in the upper gastrointestinal tract [51]. This enhanced structural accessibility clearly explains why the T4 group achieved the highest starch digestibility and IDE, whereas non-extruded diets yielded the lowest physiological values.

This enhanced enzymatic susceptibility is critical for compensating for physiological limitations in aged poultry flocks. As hens age, pancreatic function and the endogenous secretion of amylase naturally decline. Consequently, digesting raw carbohydrates with highly crystalline structures imposes a significant physiological burden on late-stage laying hens [27]. Shifting the energy-intensive initial stages of starch breakdown to a mechanical extruder remarkably alleviates the physiological strain on the digestive system of aged hens. Importantly, the apparent ileal protein digestibility did not differ among the experimental groups. This stability indicates that the selected extrusion parameters effectively balanced the thermal matrix rearrangement. The process achieved optimal starch gelatinization without inducing detrimental protein cross-linking or compromising fundamental amino acid availability. However, consistent apparent digestibility values do not unconditionally guarantee amino acid integrity at the molecular level. Thermal extrusion processes can induce severe Maillard reactions and undesired disulfide cross-linking. They may also trigger specific oxidative losses of heat-sensitive sulfur-containing amino acids, such as methionine and cysteine, thereby subtly altering the final nutritional quality of the diet [52]. The specific hydrothermal conditions employed in this study appear to have successfully managed this delicate processing balance.

The superior starch digestibility of the T4 diet likely promoted a sustained influx of absorbable glucose into the portal circulation; however, peripheral blood glucose concentrations remained tightly regulated and did not differ among experimental groups. This stability is maintained by avian homeostatic mechanisms, which are primarily regulated through insulin and glucagon dynamics [53]. In poultry, the liver functions as the primary site of lipid metabolism, accounting for over 90% of *de novo* fatty acid synthesis [54,55]. It is hypothesized that a rapid hepatic clearance and conversion of dietary carbohydrates may have contributed to the significant increases in serum triglycerides and total cholesterol observed in these hens. Importantly, this elevation in circulating lipids occurred without altering the steady-state blood glucose concentrations. To sustain continuous daily egg formation, the avian liver packages these newly synthesized lipids into very-low-density lipoproteins (VLDL) for ovarian uptake [56]. Although hepatic lipogenesis, VLDL production, and specific metabolic fluxes were not directly quantified in this study using isotope tracers or gene expression assays, we hypothesize that the elevated circulating lipids and corresponding increases in yolk weight could be linked to accelerated hepatic lipid synthesis and dynamic VLDL transport, though these physiological mechanisms remain unconfirmed.

In hens fed the completely non-extruded substitute diet (T2), the preserved crystalline structure of raw starch resists enzymatic hydrolysis in the proximal intestine, delaying starch digestion. It is possible that this resistance allows partially undigested starch to flow into the ceca. The paired ceca function as specialized bioreactors for core avian gut microbes to degrade resistant carbohydrates into short-chain fatty acids and gases [57]. This influx of fermentable substrate might hypothetically explain the significantly increased total VFA concentrations detected in the T2 group. However, the specific acid profile reveals a crucial observation. Although the absolute total VFA concentrations increased significantly, the relative proportions of individual acids did not differ among the experimental groups. These included acetic, propionic, butyric, isobutyric, valeric, and isovaleric acids. Without direct assessment of the microbiota composition via 16S rRNA sequencing, definitive conclusions regarding microbial population shifts cannot be drawn. Nevertheless, the uniform VFA profile observed in this study may demonstrate a non-specific microbial fermentation rather than a targeted prebiotic modulation of specific bacterial taxa. Concurrently, within the proximal intestine, this delayed digestion, compounded by the presence of residual HCN, could potentially contribute to the flattened mucosal appearance and blunted jejunal architecture observed in these birds, which might reflect impaired enterocyte function. Conversely, the thermomechanical extrusion process effectively mitigates these adverse effects by gelatinizing the starch matrix and eliminating volatile anti-nutritional factors. This modification facilitates rapid enzymatic cleavage and nutrient assimilation, providing readily available energy that drives the robust structural development of the jejunal mucosa. Consequently, hens fed the extruded analogs exhibit a significantly higher VH:CD ratio. An elevated VH:CD ratio is a critical biomarker of robust intestinal health, signifying an expanded surface area for maximal absorptive capacity alongside a reduced metabolic cost for continuous epithelial renewal. This optimized mucosal architecture ensures highly efficient nutrient capture, directly supporting the superior productive performance observed in the fully substituted T4 group.

Compared with the standard yellow corn control, the inclusion of extruded corn analogs (T3 and T4) sustained productive performance. While hen-day egg production and feed intake remained unchanged across all treatments, hens fed the completely substituted T4 diet achieved a significantly greater average egg weight than those on the control diet. Furthermore, both egg mass and FCR in the extruded groups closely mirrored those of the control, contrasting sharply with the diminished performance observed in the non-extruded T2 group. These results indicate that replacing conventional yellow corn with appropriately extruded cassava-based analogs preserves essential laying parameters and primarily prevents the performance decline associated with non-extruded analogs. It is hypothesized that the sustained production performance and egg quality observed in the T4 group relative to the T2 groupstems from these coordinated physiological adaptations. The extrusion process not only maximizes starch digestibility but also expands intestinal absorptive capacity. We hypothesize that this enhanced absorption might sequentially stimulate hepatic lipogenesis to support optimal egg formation, although this pathway remains speculative as specific metabolic fluxes were not directly quantified. In addition, it is hypothesized that the improved FCR may be associated with the enhanced jejunal morphology. Evaluating specific histological parameters, such as VH and CD, remains a widely recognized strategy for optimizing commercial poultry performance [58,59].

Furthermore, differences in production performance and physiological responses between the non-extruded (T2) and extruded treatments (T4) cannot be attributed exclusively to extrusion-induced changes in starch structure or improved digestibility. Residual HCN levels diverged significantly between the non-extruded T2 (41.50 ppm) and extruded T4 (2.30 ppm) diets, acting as an important confounding factor that directly impacts biological functions. Although HCN-induced amino acid depletion and its direct contribution to performance were not measured in this study, the known physiological cost of continuous HCN detoxification may have played a role in the observed differences. The intense heat and mechanical shear of the extrusion process effectively reduced the dietary HCN concentration in the extruded diets. Specifically, these thermomechanical forces cause cellular disruption and thermal degradation, while the sudden pressure drop upon exiting the die facilitates rapid volatilization of residual HCN. Ultimately, the performance of the T4 group stems from a distinct physiological advantage. Reduced HCN exposure and improved starch digestibility should be considered as possible contributors to the optimized nutrient utilization, whose individual effects cannot be separated by this experimental design. This study provides compelling empirical evidence for using cassava-based corn analogs. Our measured parameters demonstrate that replacing yellow corn at levels up to 100% increases starch digestibility and substantially reduces dietary HCN concentrations compared to non-extruded analogs. Although environmental benefits were not directly measured through life-cycle assessments, emissions, or land use metrics in this study, we propose that this nutritional innovation holds the potential to diversify feed ingredient sourcing and may help mitigate some of the supply chain vulnerabilities associated with traditional crop production.

## 5. Conclusions

The complete replacement of standard yellow corn with extruded cassava-based analogs maintains most productive parameters at levels comparable to a conventional corn diet, while effectively preventing the performance decline observed with non-extruded analogs. Thermomechanical extrusion markedly increased starch gelatinization. This improved apparent ileal starch and energy digestibility and some jejunal morphological indices. Consequently, productivity such as average egg weight, egg mass, yolk weight, and FCR differed among treatments, showing improvement in the completely substituted group relative to the non-extruded treatment. Furthermore, the extrusion parameters may support long-term feed stability by substantially reducing water activity. Adopting this nutritional strategy represents a viable alternative that preserves laying hen productivity while potentially diversifying global feed ingredient sourcing.

## Figures and Tables

**Table 1:** Ingredient formulation and analyzed chemical composition of the corn analogs.

Item	Yellow Corn	Corn Analogs	
		Non-Extrusion	Extrusion
Ingredients (% as-fed basis)			
Cassava chip	-	60.00	60.00
Broken rice	-	22.40	22.40
Corn gluten meal (60% CP)	-	13.50	13.50
Rice bran oil	-	4.00	4.00
Canthaxanthin (10%)	-	0.10	0.10
Total	-	100.00	100.00
Analyzed chemical composition ^1^ (% as-DM basis)			
Dry matter	88.50 ± 0.38	88.62 ± 0.32	89.35 ± 0.45
Crude protein	8.85 ± 0.15	8.82 ± 0.12	8.80 ± 0.10
Ether extract	4.10 ± 0.10	4.12 ± 0.08	4.08 ± 0.07
Crude fiber	2.35 ± 0.08	2.30 ± 0.10	2.25 ± 0.09
Crude ash	1.40 ± 0.04	1.45 ± 0.05	1.48 ± 0.04
Nitrogen-free extract	83.30 ± 0.32	83.31 ± 0.30	83.34 ± 0.28
Gross energy (kcal/kg)	4352 ± 22.00	4348 ± 20.00	4355 ± 18.00

^1^ Proximate compositions, including crude protein, crude ash, crude fiber, and ether extract, were determined according to the standard procedures of AOAC [29]. The gross energy was determined using an automatic bomb calorimeter (AC-500, LECO Corporation, St. Joseph, MI, USA) calibrated against a benzoic acid standard. All analyzed chemical compositions were determined in triplicate per sample (*n* = 3), and values are expressed as means ± standard deviation.

**Table 2:** Feed formulations and analyzed nutrient profiles of the experimental diets.

Item	T1	T2	T3	T4
Feed ingredients (%)				
Yellow corn	50.0	0.0	25.0	0.0
Extruded Corn Analogs	0.0	0.0	25.0	50.0
Non-extruded Corn Analogs	0.0	50.0	0.0	0.0
Broken rice	8.3	8.3	8.3	8.3
Rice bran	5.0	5.0	5.0	5.0
Soybean meal (48% CP)	18.3	18.3	18.3	18.3
Fish meal (60% CP)	3.0	3.0	3.0	3.0
Porcine meat meal (50% CP)	3.0	3.0	3.0	3.0
Palm oil	2.0	2.0	2.0	2.0
Calcium Carbonate	8.0	8.0	8.0	8.0
Dicalcium phosphate (18% P)	1.5	1.5	1.5	1.5
Vitamin-mineral premix ^1^	0.5	0.5	0.5	0.5
L-Lysine	0.2	0.2	0.2	0.2
DL-Methionine	0.2	0.2	0.2	0.2
Total	100.0	100.0	100.0	100.0
Analyzed chemical composition ^2^				
Crude protein (%)	17.51	17.49	17.52	17.50
Crude ash (%)	12.62	12.85	12.74	12.85
Crude fiber (%)	2.39	2.59	2.45	2.54
Ether extract (%)	5.50	5.54	5.52	5.55
Hydrogen Cyanide (ppm)	ND	41.50	1.18	2.30
Gross energy (kcal/kg)	3696	3693	3697	3695
Calculated metabolizable energy (kcal/kg)	2815	2815	2815	2815

T1, a standard corn-based control; T2, 100% replaced with non-extruded corn analogs; T3, 50% replaced with extruded corn analogs; T4, 100% replaced with extruded corn analogs; ND, not detected. ^1^ The vitamin-mineral premix supplied the following per kilogram of diet: vitamin A (trans-retinyl acetate), 12,000 IU; vitamin D_3_ (cholecalciferol), 3000 IU; vitamin E (all-rac-tocopherol acetate), 12 mg; vitamin K_3_ (menadione bisulfate complex), 3.6 mg; vitamin B_2_, 5.4 mg; vitamin B_6_, 4.2 mg; vitamin B_1_, 1.4 mg; vitamin B_12_ (cyanocobalamin), 0.02 mg; choline chloride, 210 mg; biotin, 45 mg; pantothenic acid, 9 mg; nicotinic acid, 9 mg; folic acid, 0.6 mg; zinc sulfate, 60 mg; manganese oxide, 96 mg; iron, 54 mg; and copper sulfate, 12 mg; selenium, 0.18 mg; iodine, 1.08 mg; cobalt, 0.3 mg; ^2^ Proximate compositions including crude protein, crude ash, crude fiber, and ether extract, as well as hydrogen cyanide content, were determined according to the standard procedures of AOAC [29]. The gross energy was determined using an automatic bomb calorimeter (AC-500, LECO Corporation, St. Joseph, MI, USA) calibrated against a benzoic acid standard. All analyzed chemical compositions were determined in triplicate per sample (*n* = 3).

**Table 3:** Physicochemical properties, color profiles, and water activity of yellow corn and both non-extruded and extruded corn analogs.

Item	Yellow Corn	Corn Analogs		Pooled SEM	*p*-Value
		Non-Extrusion	Extrusion		
Physicochemical properties					
Degree of gelatinization (%)	14.20 ^b^	16.80 ^b^	88.50 ^a^	6.48	<0.001
Water absorption index (g/g)	2.25 ^b^	2.41 ^b^	5.65 ^a^	0.30	<0.001
Water solubility index (%)	4.12 ^b^	4.35 ^b^	16.80 ^a^	1.14	<0.001
Color profiles					
Lightness (L*)	65.50	66.11	64.8	0.72	0.770
Redness (a*)	4.80	4.65	4.95	0.20	0.840
Yellowness (b*)	38.52	39.10	37.8	0.89	0.845
Water activity	0.58 ^a^	0.56 ^a^	0.42 ^b^	0.01	<0.001

^a,b^ Different superscripts in the same row indicate significant difference (*p* < 0.05).

**Table 4:** Productive performance and egg quality of late-phase laying hens fed diets replacing standard yellow corn with extruded corn analogs.

Item	T1	T2	T3	T4	Pooled SEM	*p*-Value
Initial body weight (kg)	1.85	1.84	1.85	1.86	0.01	0.850
Initial egg production (%)	85.20	84.90	85.50	85.40	0.23	0.802
Final body weight (kg)	1.95	1.92	1.94	1.96	0.01	0.487
Productivity parameters						
Hen-day egg production (%)	76.46	75.28	76.87	77.09	0.84	0.883
Average daily feed intake (g/hen)	105.35	103.66	105.42	105.02	1.59	0.979
Average egg weight (g/egg)	61.51 ^b^	58.92 ^c^	62.22 ^b^	63.55 ^a^	0.28	<0.001
Egg mass (g/hen/d)	47.01 ^ab^	44.31 ^b^	47.82 ^ab^	48.99 ^a^	0.55	0.017
Feed conversion ratio	2.24 ^ab^	2.34 ^a^	2.20 ^ab^	2.14 ^b^	0.02	0.014
Egg quality						
Whole egg weight (g)	61.90	59.10	62.70	63.50	0.71	0.141
Yolk weight (g)	15.50 ^ab^	15.00 ^b^	16.60 ^ab^	17.50 ^a^	0.32	0.020
Albumen weight (g)	39.70	37.50	39.40	39.20	0.42	0.247
Shell weight (g)	6.70	6.60	6.70	6.80	0.06	0.731
Albumen height (mm)	8.20	7.95	8.30	8.45	0.08	0.178
Shell thickness (mm)	0.38	0.36	0.38	0.39	0.01	0.113
Shell strength (N)	41.50	39.80	41.20	42.10	0.36	0.132
Yolk height (mm)	18.50	17.80	18.40	18.60	0.15	0.258
Yolk diameter (mm)	40.20	40.00	40.30	40.10	0.10	0.769
Yolk index	0.46	0.44	0.46	0.46	0.01	0.278
Yolk color	11.20	11.00	11.30	11.20	0.09	0.676
Haugh unit	90.09	89.46	90.42	91.00	0.45	0.686

T1, a standard corn-based control; T2, 100% replaced with non-extruded corn analogs; T3, 50% replaced with extruded corn analogs; T4, 100% replaced with extruded corn analogs; SEM, standard error of the mean. ^a,b,c^ Different superscripts in the same row indicate significant differences (*p* < 0.05).

**Table 5:** Digestible energy and apparent ileal nutrient digestibility in late-phase laying hens fed diets replacing standard yellow corn with extruded corn analogs.

Item ^1^	T1	T2	T3	T4	Pooled SEM	*p*-Value
Ileal digestible energy (kcal/kg)	2990 ^ab^	2950 ^b^	3009 ^ab^	3063 ^a^	14.69	0.038
Apparent ileal digestibility (%)						
Starch	88.3 ^ab^	85.2 ^b^	87.4 ^ab^	92.1 ^a^	0.83	0.015
Energy	80.9 ^ab^	79.9 ^b^	81.4 ^ab^	82.9 ^a^	0.40	0.041
Protein	84.5	80.8	83.4	81.2	0.83	0.354

T1, a standard corn-based control; T2, 100% replaced with non-extruded corn analogs; T3, 50% replaced with extruded corn analogs; T4, 100% replaced with extruded corn analogs; SEM, standard error of the mean. ^1^ All values are expressed on a dry-matter basis. ^a,b^ Different superscripts in the same row indicate significant differences (*p* < 0.05).

**Table 6:** Blood glucose concentrations and serum biochemical parameters of late-phase laying hens fed diets replacing standard corn with extruded corn analogs.

Item	T1	T2	T3	T4	Pooled SEM	*p*-Value
Blood glucose (mg/dL)	195.8	197.4	201.0	198.9	0.80	0.123
Serum biochemical parameters						
Total protein (g/dL)	5.2	5.0	5.3	5.4	0.15	0.389
Albumin (g/dL)	2.1	2.0	2.1	2.2	0.05	0.574
Globulin (g/dL)	3.1	3.0	3.2	3.2	0.06	0.654
Creatinine (mg/dL)	0.4	0.5	0.3	0.6	0.07	0.419
Uric acid (mg/dL)	4.8	5.1	4.7	4.6	0.13	0.580
Total cholesterol (mg/dL)	152.0 ^ab^	145.0 ^b^	168.0 ^ab^	182.0 ^a^	4.86	0.029
Triglyceride (mg/dL)	780.0 ^ab^	750.0 ^b^	860.0 ^a^	840.0 ^ab^	14.46	0.020
Aspartate aminotransferase (U/L)	155.0	188.0	160.0	152.0	6.70	0.210
Alanine aminotransferase (U/L)	18.0	22.0	19.0	17.0	0.81	0.146

T1, a standard corn-based control; T2, 100% replaced with non-extruded corn analogs; T3, 50% replaced with extruded corn analogs; T4, 100% replaced with extruded corn analogs; SEM, standard error of the mean. ^a,b^ Different superscripts in the same row indicate significant differences (*p* < 0.05).

**Table 7:** Cecal VFA profiles of late-phase laying hens fed diets replacing standard yellow corn with extruded corn analogs.

Item (µmol/g)	T1	T2	T3	T4	Pooled SEM	*p*-Value
Acetic acid	60.5	65.1	59.0	57.5	1.20	0.120
Propionic acid	15.2	16.5	17.0	14.5	0.54	0.349
Butyric acid	14.5	12.2	15.5	16.8	0.74	0.152
Isobutyric acid	1.5	1.9	1.6	1.4	0.09	0.290
Valeric acid	1.5	1.2	1.4	1.5	0.07	0.421
Isovaleric acid	1.8	2.3	1.9	1.6	0.12	0.217
Total VFA	95.0 ^ab^	99.2 ^a^	96.4 ^ab^	93.3 ^b^	0.80	0.045

T1, a standard corn-based control; T2, 100% replaced with non-extruded corn analogs; T3, 50% replaced with extruded corn analogs; T4, 100% replaced with extruded corn analogs; SEM, standard error of the mean; VFA, volatile fatty acid. ^a,b^ Different superscripts in the same row indicate significant differences (*p* < 0.05).

**Table 8:** Intestinal morphological alterations of late-phase laying hens fed diets replacing standard yellow corn with extruded corn analogs.

Item	T1	T2	T3	T4	Pooled SEM	*p*-Value
Duodenum						
Villus height (µm)	1220.00	1180.00	1200.00	1230.00	11.44	0.450
Villus surface area (mm^2^)	0.42	0.40	0.41	0.43	0.01	0.678
Crypt depth (µm)	185.00	195.00	188.00	182.00	2.53	0.320
VH:CD	6.62	6.08	6.42	6.79	0.14	0.336
Jejunum						
Villus height (µm)	965.00 ^a^	845.00 ^b^	940.00 ^ab^	985.00 ^a^	18.20	0.018
Villus surface area (mm^2^)	0.32	0.30	0.33	0.34	0.01	0.262
Crypt depth (µm)	170.00 ^ab^	195.00 ^a^	175.00 ^ab^	165.00 ^b^	4.17	0.042
VH:CD	5.72 ^a^	4.35 ^b^	5.42 ^ab^	6.05 ^a^	0.21	0.013
Ileum						
Villus height (µm)	630.00	610.00	625.00	640.00	6.02	0.380
Villus surface area (mm^2^)	0.20	0.19	0.21	0.21	0.01	0.710
Crypt depth (µm)	140.00	145.00	142.00	138.00	1.56	0.460
VH:CD	4.52	4.22	4.42	4.65	0.09	0.379

T1, a standard corn-based control; T2, 100% replaced with non-extruded corn analogs; T3, 50% replaced with extruded corn analogs; T4, 100% replaced with extruded corn analogs; SEM, standard error of the mean. ^a,b^ Different superscripts in the same row indicate significant difference (*p* < 0.05).

## Data Availability

The data are contained in the manuscript.

## Abbreviations

The following abbreviations are used in this manuscript:

AID	Apparent ileal digestibility
CD	Crypt depth
FCR	Feed conversion ratio
IDE	Ileal digestible energy
PBS	Phosphate-buffered saline
VFAs	Volatile fatty acids
VH	Villus height
VLDL	Very-low-density lipoproteins
WAI	Water absorption index
WSI	Water solubility index
